# Reconstructive procedures for segmental resection of bone in giant cell tumors around the knee

**DOI:** 10.4103/0019-5413.32044

**Published:** 2007

**Authors:** Aditya N Aggarwal, Anil K Jain, Sudhir Kumar, Ish K Dhammi, Bhagwat Prashad

**Affiliations:** Department of Orthopedics, University College of Medical Sciences and GTB Hospital, Shahadara, Delhi - 110 095, India

**Keywords:** Enbloc resection, giant cell tumor, reconstruction of knee

## Abstract

**Background:**

Segmental resection of bone in Giant Cell Tumor (GCT) around the knee, in indicated cases, leaves a gap which requires a complex reconstructive procedure. The present study analyzes various reconstructive procedures in terms of morbidity and various complications encountered.

**Materials and Methods:**

Thirteen cases (M-six and F-seven; lower end femur-six and upper end tibia -seven) of GCT around the knee, radiologically either Campanacci Grade II, Grade II with pathological fracture or Grade III were included. Mean age was 25.6 years (range 19-30 years).

Resection arthrodesis with telescoping (shortening) over intramedullary nail (n=5), resection arthrodesis with an intercalary allograft threaded over a long intramedullary nail (n=3) and resection arthrodesis with intercalary fibular autograft and simultaneous limb lengthening (n=5) were the procedure performed.

**Results:**

Shortening was the major problem following resection arthrodesis with telescoping (shortening) over intramedullary nail. Only two patients agreed for subsequent limb lengthening. The rest continued to walk with shortening. Infection was the major problem in all cases of resection arthrodesis with an intercalary allograft threaded over a long intramedullary nail and required multiple drainage procedures. Fusion was achieved after two years in two patients. In the third patient the allograft sequestrated. The patient underwent sequestrectomy, telescoping of fragments and ilizarov fixator application with subsequent limb lengthening. The patient was finally given an ischial weight relieving orthosis, 54 months after the index procedure.

After resection arthrodesis with intercalary autograft and simultaneous lengthening the resultant gap (∼15cm) was partially bridged by intercalary nonvascularized dual fibular strut graft (6-7cm) and additional corticocancellous bone graft from ipsilateral patella. Simultaneous limb lengthening with a distal tibial corticotomy was performed on an ilizarov fixator. The complications were superficial infection (n=5), stress fracture of fibula (n=2). The stress fracture fibula required DCP fixation and bone grafting. The usual time taken for union and limb length equalization was approximately one year.

**Conclusion:**

Resection arthrodesis with intercalary dual fibular autograft and cortico-cancellous bone grafting with simultaneous limb lengthening achieved limb length equalization with relatively short morbidity.

Giant cell tumor (GCT) most frequently occurs in the distal end of the femur and the proximal end of the tibia. The majority of patients are between 20 and 45 years of age.^1^

A variety of treatment modalities are available for GCT. They include curettage and bone grafting, cryotherapy, phenol application, insertion of methylmethacrylate, insertion of hydroxyapatite, resection followed by allograft or prosthetic reconstruction.

Intralesional procedures have very high local recurrence while wide or radical procedures few recurrence.^1^ However, wide/radical resection requires reconstructive procedures. The present study evaluates various reconstructive options after en bloc resection for GCT around the knee to evolve a biologically sound, reconstructive procedure with reduced morbidity.

## MATERIALS AND METHODS

Thirteen cases of GCT around the knee presenting between 1988 and 2003 were included in the study. All the cases included were radiographically either Campanacci Grade II, Grade II with pathological fracture or Grade III.

There were six males and seven females in the study group. The mean age was 25.6 years (range 19-30 years). The distal end of the femur was involved in six cases and the upper end of the tibia was involved in seven cases. Two cases had additional findings of aneurysmal bone cyst. Twelve patients were fresh cases of GCT while one patient presented with recurrence and infection after having an initial curettage and bone grafting procedure done elsewhere for GCT of lower end femur. All the cases were operated by two senior authors (SK, AKJ). The segmental resection of tumor bone was done. The various reconstructive procedures used to bridge the bone gap in our series were shortening over intramedullary nail (telescoping) (n=five), resection arthrodesis with intercalary allograft (n=three), resection arthrodesis with intercalary autograft (n=five) and combining distraction histogenesis with intercalary autograft to attain limb length after resection arthrodesis.

Resection arthrodesis with shortening (telescoping) was performed in five cases. Four patients underwent segmental resection of the bone and telescoping over an intramedullary nail. Out of these four patients, only one patient [Figure 1] who had residual shortening of 12 cm agreed for limb lengthening. One year later the nail was removed and the tibia was lengthened 10 cm by a ring external fixator. In the fifth patient (Campanacci Grade III) a segmental resection of limb was preformed. There was soft tissue extension with a fungating mass on the anterior aspect of the knee. The skin, soft tissue, affected lower end of the femur and knee joint were removed en bloc. At this stage the distal limb was attached to the proximal limb by neurovascular bundle only. After achieving safe margins, a gap of 20 cm was created. The distal stump was telescoped on the femur and stabilized with a prefabricated intramedullary nail [Figure 2]. The patient was immobilized in a toe to groin cast till clinico-radiological arthrodesis was achieved. This patient was taken up for subsequent lengthening with ring external fixator. The lengthening of the limb was performed in two stages, initially by tibial corticolomy (12 cm) and then by femoral corticolomy (6 cm).

**Figure 1A:**
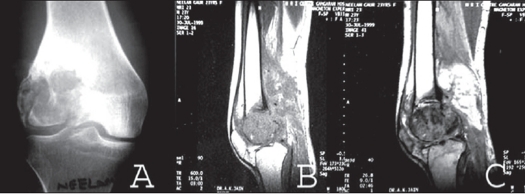
A) AP x-ray of a 19 year old female presenting with recurrence and discharging sinus following curettage and bone grafting of GCT of distal femur. B, C) MRI scan (T1W and T2W images) showing break in the cortex and adjoining soft tissue involvement.

**Figure 1B:**
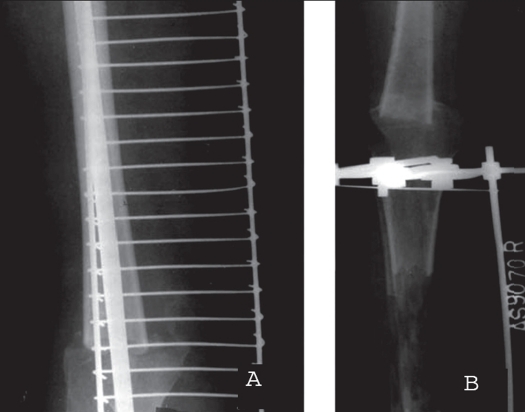
A) X-ray showing segmental resection of bone and telescoping of the fragments over an intramedullary nail. B) Tibial lengthening being performed after resection arthrodesis was achieved.

**Figure 2:**
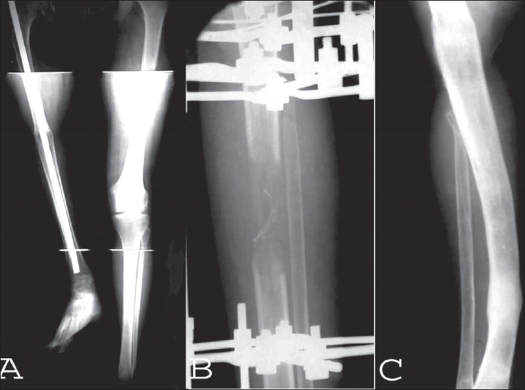
A) Combined x-ray of a patient who underwent segmental resection of limb with telescoping on an intramedullary nail. Note the marked residual shortening. B) First stage tibial lengthening being performed after nail removal. C) Final outcome of the patient after second stage lengthening

**Figure 3:**
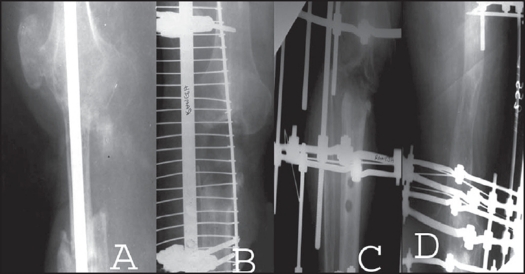
A) Postoperative x-ray of the patient showing intercalary allograft threaded over an intramedullary nail. B) Sequestered graft removed and tubular external fixator applied. C, D) Telescoping of the bone ends with simultaneous lengthening of femur

Resection arthrodesis with an intercalary allograft (decalcified and ethanol preserved) was preformed in three patients. After achieving wide margins following segmental bone resection, the defect was bridged by tubular allografts threaded over an intramedullary nail [Figure 3]. All three developed secondary infection. In two superficial infection could be controlled. In one case, the allograft sequestered due to prolonged infection.

**Figure 4:**
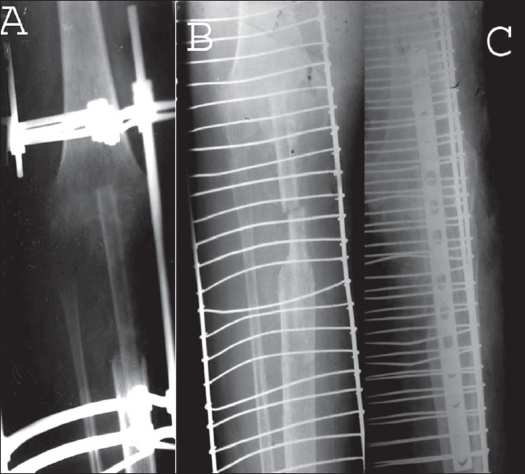
A) Resection arthrodesis with intercalary fibular grafting in GCT of proximal tibia. The ipsilateral proximal fibula was used as non-vascularized graft. B) Fracture of the nonvascularized intercalary fibular graft. C) Plating and bone grafting performed for fracture of the fibular graft

**Figure 5:**
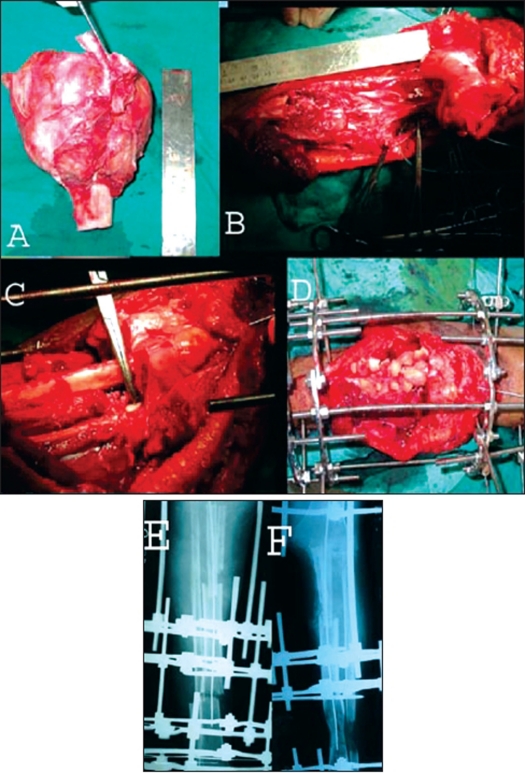
A) Resected specimen after enbloc excision of GCT of proximal tibia. B) The tumour resection leaving a gap of around 14 cm. C) The gap being partially bridged by intercalary dual fibular graft with intramedullary K-wire. D) The fibular graft being augmented by copious cortico-cancellous graft. The extremity is stabilized by ring fixator for simultaneous limb lengthening. E) Postoperative radiograph of the patient showing dual intercalary fibular graft with intramedullary K-wire. A distal tibial corticotomy has been performed for limb lengthening. F) Followup of the patient showing crossunion between the dual fibular graft. The regenerate for limb lengthening is beginning to consolidate.

In our subsequent cases (n=five) we performed resection arthrodesis with intercalary autograft. After en bloc excision of the tumor, the resultant gap (∼ 15 cm) was partially bridged by intercalary nonvascularized fibular strut graft (6-7 cm). Additional corticocancellous bone graft from ipsilateral patella was also used. The extremity was stabilized by an Ilizarov ring fixator assembly. A distal tibial corticotomy was performed for simultaneous limb lengthening. In the first two patients only a single fibular graft was used. After limb lengthening and consolidation of the regenerate, the ring fixator assembly was removed at eight and 17 months, respectively. Subsequently both the patients developed pathological fracture of intercalary fibular graft [Figure 4]. Both patients required plating and bone grafting for the pathological fracture, which subsequently united. So, the procedure was modified and dual fibular grafting with K wire stabilization of the fibula was done in the next three cases [Figure 5].

## RESULTS

The patients who underwent segmental resection of bone with shortening were ambulatory with a shoe raise. Shortening was the major problem in three cases. The arthrodesis was achieved in 12-18 months in all cases. Only two underwent lengthening subsequently. The problems encountered during lengthening were long morbidity, multiple surgical procedures, tendo-achilles contracture and toe flexor contractures which required Z-plasty, and pin tract infections. It took three years to complete the treatment.

Infection was a major problem in all the patients who underwent resection arthrodesis with an intercalary allograft (decalcified and ethanol-preserved). The arthrodesis was achieved after two years in two patients. The third patient underwent nail removal, debridement, sequestrectomy, telescoping of fragments over an AO external fixator stabilization, 27 months after the index surgery. Four months later the AO fixator was converted to ring external fixator with tibial corticotomy for simultaneous limb lengthening. The ring external fixator was removed after subsequent 23 months and the patient was given an ischial weight-relieving caliper for one year.

Three patients who underwent segmental resection of bone with intercalary dual fibular autograft with simultaneous lengthening had no major complications except for superficial pin tract infection. The mean time of fixator application in these cases was 54 weeks. The mean lengthening achieved in the last three cases was 6.3 cm.

## DISCUSSION

The World Health Organization has classified GCT as “an aggressive, potentially malignant lesion”.^2^ Its histogenesis is uncertain.^2^,^3^ Historically, curettage / intralesional excision has been associated with a high rate of recurrence (30-50%).^2^ Intralesional excision / curettage combined with local adjuvants like methylmethacrylate and liquid nitrogen (thermal action) or phenol and hydrogen peroxide (chemical action), may decrease the rates of local recurrence.^3^ However, the adequacy of the removal of tumor rather than the use of adjuvant modalities is what determines the risk of recurrence.^4^,^5^

The risk of local recurrence after an en bloc resection involving the joint is lower than that after an intralesional procedure.^4^ Campanacci reported a recurrence rate of zero in 58 wide (en bloc resections) or radical procedures.^1^

Now increasing emphasis is being laid on preservation of joint in treating GCT.^2^,^3^ Resection is usually performed in a) Stage 3 lesions, which have already destroyed the cortex and tend to recur more often; b) when the defect is large; and c) when the joint surface is destroyed or cannot be salvaged. However, Szendroi tries to preserve the joint even in Stage 3 lesion, taking into account the higher probability of recurrence. He believes that extended curettage and application of bone cement are the most accepted methods of treatment of GCT.^2^ When the tumor is less than 1 cm from the articular surface, the incidence of degenerative changes in articular cartilage after the use of cement alone is more than 2.5 times greater than that when the tumor is more than 1 cm away.^6^ Interposing a cm or two of bone graft between the cartilage and cement may reduce heat damage and the resultant early degenerative changes.^6^ Studies have shown that cement constructs are less rigid than normal subchondral bone or successful bone graft.^3^ Our patients generally presented late in the course of the disease when the lesion had become large in size and abutting the articular cartilage despite being Campanacci Grade 2 lesion. Most of our cases are from the last two decades and hence we have used various methods to reconstruct the gap.

Gitelis et al compared the results of en bloc resection of GCT (n=20) and intralesional excision with adjunctive local insertion of methylmethacrylate or phenol (n=20). They reported only one recurrence in the intralesional surgery group. There were no recurrences in the patients who had an en bloc resection. However, the disadvantage of this treatment was the relatively poor functional outcome. The limitation of this study was that only Campanacci Stage II tumors around the knee were subjected to intralesional surgery^7^ (curettage + phenol +methylmethacrylate / bone graft) while in the resection group they had Stage III lesion also.

An arthrodesis is less attractive initially but once it is achieved it provides a stable leg and the patient is unlikely to require revision surgery. A realistic estimate of the expected function after a proposed reconstructive procedure must be given preoperatively. Resection arthrodesis with shortening provided a stable extremity, but with unacceptable shortening. A patient refusing subsequent lengthening procedures is probably an indicator of the limited resources available to the patient in the developing world. Although the function of the extremity was compromised, the emotional acceptance of the residual deficit was good due to preoperative counseling of the patients in our series. In most developing countries resection-shortening-distraction offers a very real alternative. In a young active patient with GCT around the knee, with a normal lifespan, endoprosthesis is not a sound biological solution that matches life expectancy. It is likely to require multiple revision surgeries.

The limited availability of autogenous bone has led to the interest in the use of allograft for arthrodesis. The major concern about allograft is the high complication rate.^8^,^9^ These include infection, fracture and nonunion.^8^ In our patients, infection was the major problem.

Another method of reconstruction after en bloc resection is the use of intercalary autograft. Enneking and Shirly reported 20 cases of local resection and arthrodesis employing an intramedullary nail and autogenous segmental cortical grafts obtained from the same extremity. The indication for selection of the procedure was a lesion in the epiphyseal region of the femur or tibia in such a way that adequate resection with preservation of joint function was not possible. They continued external support for a period of one year if union occurred. They claimed 95% good functional results at the end of two years from surgery by this method. Four patients had a nonunion while four patients had spontaneous fatigue fractures of their grafts. All sites of nonunion subsequently healed four to 11 months after supplementary iliac bone grafting.^10^

Yadav advocates the use dual fibular graft to bridge the intercalary defect after en bloc resection.^13^ He reported 52 patients (GCT-37; osteosarcoma-15) where the size of gap ranged from 9-24 cm. In the later part of his series he advocates the use of Kirschner wires inside the long grafts to help in maintaining the continuity of the graft when a stress fracture occurs.^13^

In the last 15 years distraction histogenesis is in vogue. Resection-shortening-distraction offers a very good alternative. However, in large defects the lengthening and consolidation time can be substantial.^11^ We have used intercalary autogenous nonvascularized fibular graft to partially bridge the defect and achieve simultaneous residual limb lightening by a distal tibial corticotomy.

Kapukaya *et al* described limb reconstruction with the callus distraction method in seven cases of tumors of the distal femur. The defect after tumor resection ranged from 8-20 cm.^11^ Tsuchiya *et al* described the use of the Illizarov technique for management of subarticular defects after en bloc resection or curettage and phenol cauterization in GCT of the proximal tibia in five patients. The mean length of bone defect was 5.7 cm and the mean duration of external fixation was 233 days. The advantages of this method include the lack of graft rejection, the reattachment of ligaments and tendon to the bone, the prevention of articular collapse, early movement of the knee and ankle joint and early weight bearing. Disadvantages include the long duration of external fixator application, pin tract infection, wire breakage and frustration of patients due to long duration of treatment.^12^ However, most of our patients present to us late when the subchondral bone plate is thinned or breached and the resultant defect after en bloc excision is large. Bridging such a large gap by distraction histogenesis alone is a lengthy process and has its own sets of problems and complications.

Resection arthrodesis with intercalary dual fibular grafts with intramedullary K-wires with cortico-cancellous graft and simultaneous distraction histogenesis may provide a good biological long-term alternative solution for GCT around the knee. It helped us in achieving a functional limb with a sound arthrodesis in a reasonably short duration of time.
